# Examining the Experiences of Adult Learners in Higher Education

**DOI:** 10.1093/geroni/igab046.2829

**Published:** 2021-12-17

**Authors:** Jyotsna Kalavar, Kirsten Magda, Raquel Ariyo

**Affiliations:** 1 Georgia Gwinnett College, Lawrenceville, Georgia, United States; 2 Georgia Gwinnett College, Georgia Gwinnett College, Georgia, United States

## Abstract

In the last decade, there has been a shift of more non-traditional adult learners returning to pursue undergraduate education. Though traditional age students are in the majority, a rising population of adult learners has been steadily increasing. They are typically students who are 25 years and older, attend part-time, work full-time, and tend to juggle family or dependent demands with schoolwork. Studies show that these adult learners are at high-risk for academic underachievement and dropping out. However, educational institutions still operate with the same traditional learning paradigm that they previously used (without acknowledging the wealth of life experiences that adult learners bring), leading us to the question of how adult learners perceive their academic learning experiences. In this study, 171 adult learners (students aged 25 and older at the time of matriculation) at a regional college in Atlanta, participated in an online survey that examined their academic experiences, specifically meaningfulness of coursework, course delivery approaches, and the advantages as well as disadvantages of being an adult learner. Majority were females (83%), and the ethnic breakdown was as follows: White (41%), African American (26%), Hispanic (12%), and 22% reported other. Results of this study indicate that academic institutions need to pay attention to the learning experiences of this burgeoning student population. Understanding their perspectives on their academic experiences hold major implications for long-term meaningful change in academia.

